# Phenotyping myocardial injury related to COVID and SARS-CoV-2 vaccination: insights from cardiovascular magnetic resonance

**DOI:** 10.3389/fcvm.2023.1186556

**Published:** 2023-06-15

**Authors:** Ria Garg, Muzna Hussain, Matthias G. Friedrich

**Affiliations:** ^1^ Department of Internal Medicine, Geisinger Wyoming Valley Hospital, Wilkes Barre, PA, United States; ^2^ Department of CV Imaging, Courtois CMR Research Group at the Research Institute of the McGill University Health Centre, Montreal, Canada; ^3^ Division of Experimental Medicine, Departments of Medicine and Diagnostic Radiology, Universitaire de Santé McGill Site Glen, Montreal, QC, Canada

**Keywords:** COVID-19, COVID vaccine, CMR (cardiovascular magnetic resonance), myocardial injury, phenotype

## Introduction

Myocardial injury has been reported in patients with acute or previous COVID-19 and in relation to the SARS-CoV-2 vaccine (1). While infrequent, cardiac involvement has been associated with an impaired outcome (2). Several different mechanisms have been proposed in the literature for each entity. As the prime non-invasive diagnostic procedure for myocardial inflammation, cardiovascular magnetic resonance imaging (CMR) has played an essential role during the pandemic in diagnosing associated myocardial inflammation, having a similar diagnostic accuracy compared to invasive endomyocardial biopsy (3). There has been an extensive body of literature on CMR findings in all these syndromes that showed different patterns of myocardial pathology such as global myocardial edema, irreversible myocardial injury such as necrosis and scarring, as well as pericardial effusion. Several such studies have reported similarities of such patterns to those found in myocardial inflammation triggered by other viruses (4, 5), albeit with some difference in some studies (6, 7).

We aim to summarize the CMR findings and discuss areas of similarity.

## Myocardial injury in acute COVID-19

### Prevalence

Though the most virulent manifestation of COVID-19 is acute respiratory distress syndrome, cardiac injury reflected through elevated troponin concentrations has been increasingly reported (8, 9). Various studies have reported an overall prevalence of acute myocardial injury ranging 5%–38% (10). One study described myocardial injury prevalence of 36% with significant association with death and a higher troponin-I associated with a higher risk of death (11).

### Clinical presentation

Myocardial injury is typically characterized by chest pain, dyspnea and palpitations, with or without elevated cardiac biomarkers such as high-sensitivity cardiac troponin (hsTn) and/or creatinine kinase MB (12). Notably, electrocardiographic and echocardiographic findings in patients with COVID-19-related myocardial injury can be normal (13).

### Proposed mechanisms

Several etiologies have been proposed to cause myocardial injury in COVID-19, including direct viral injury, pre-existing chronic injury, supply-demand imbalance, multi-organ failure (14–16), stress-induced cardiomyopathy Takotsubo, or plaque rupture with ischemic events (16–18).

Infection with SARS-CoV-2 impairs endothelial function and hemostatic balance, increases thrombin activity, reduces plasminogen activator inhibitor-1 activity and accelerates the production of fibrin degradation products (19). Endothelial inflammation with vascular edema and disseminated intravascular coagulation may lead to microvascular dysfunction. These factors may exacerbate myocardial oxygen supply/demand imbalance due to hypoxia and tachycardia (20).

### Diagnostic utility of CMR

CMR is unique in its capability to non-invasively characterize myocardial inflammation and injury. It is considered the gold standard imaging modality in diagnosing myocarditis (21). The CMR criteria for assessing myocardial inflammation (“Lake Louise Criteria”) include a high myocardial signal intensity in T2-weighted images indicating myocardial edema, increased early uptake of gadolinium with a high signal intensity in T1-weighted images (early gadolinium enhancement) as an indicator for hyperemia and capillary leakage, and a high signal intensity in late gadolinium enhancement (LGE) demonstrating necrosis or scar. Based on these criteria, CMR can identify myocardial damage with a sensitivity of 78% and specificity of 88% [AUC 0.83 (0.79–0.86)] (22). In 2018, T1 mapping and T2 mapping were included (3), leading to a similar or higher accuracy (75%–91.8%) as the original criteria (23, 24). The Lake Louise Criteria have significant value in guiding patient management (25) and are also part of the recommendations for using CMR in COVID-19 (26).

### Pertinent CMR findings

Myocardial necrosis and scar assessed by LGE imaging are associated with an impaired outcome, while LGE-negative patients have an excellent prognosis, regardless of symptoms (27, 28). Myocarditis-induced injury is typically localized sub-epicardial and/or intramurally, frequently in the basal to mid-inferolateral segments (29). Myocardial edema as an invariable component of active inflammation can be visualized by native T2 mapping. This technique can verify active inflammation with a sensitivity of 89%. Native T1 mapping is also sensitive to myocardial injury and edema and thus can be used to visualize tissue inflammation (30).

## Myocardial injury in post-COVID syndrome

### Prevalence

While most patients with mild-moderate disease recover within 2 weeks, there is a percentage of the population which do not return to baseline even after 14–21 days (31). The prevalence of post-COVID-19 myocardial injury remains uncertain, with reported data ranging from 0.5% to 20% (32–35). Recent data indicate that as much as 7% of COVID-19-related mortalities may be attributable to myocarditis (36).

### Clinical presentation

The Royal College of General Practitioners in the UK has divided COVID-19 infection into: (1) Acute COVID-19 (within 4 weeks after disease onset), (2) Ongoing Symptomatic COVID-19 (persisting for 4–12 weeks), and (3) Post-COVID-19 Syndrome (or Long COVID), if persistent after 12 weeks (32). Persistent chest pain has been reported in up to 20% of COVID-19 survivors over a 2-month follow-up and recurrent palpitations in up to 9% after 6 months, with shortness of breath reported in up to 8% (37).

### Proposed mechanisms for post-COVID-19 syndromes

The underlying pathophysiology is poorly understood, including a systemic inflammatory response with cytokine storm, counter-balanced by a compensatory anti-inflammatory response syndrome to prevent widespread multiorgan dysfunction (38) as well as virus persistence and latent virus reactivation of SARS-COV-2. For long COVID, adrenal insufficiency and cerebral dysregulation have been discussed.

### Diagnostic utility of CMR

CMR in COVID-19-related cardiac injury is highly mandated and rapidly growing, due to its ability not only to diagnose acute and chronic sequelae of myocardial inflammation but also to provide a more detailed understanding of the pathophysiological phenomenon behind cardiac involvement and differentiate them from other various pathological etiologies (39).

In patients that have recovered from COVID-19, the reported incidence of myocardial inflammation varies greatly, ranging from 2.4% to 30% (2), in one controversial paper even 78%. Non-ischemic scar patterns among participants suggest a non-ischemic cause of cardiac injury (40, 41). One study reported lower left ventricular ejection fraction, higher left ventricular volumes, higher native T1 values consistent with myocardial edema or interstitial fibrosis (42), and high T2 values suggesting myocardial edema (43) when compared with healthy control subjects and risk factor–matched control subjects. These findings correlated with higher levels of hsTn and active lymphocytic inflammation on endomyocardial biopsy specimens (41). Native T1 and T2 mapping provided the best discriminatory ability to detect COVID-19-associated myocardial disease (41).

CMR may play an important role not only during pandemics but also afterwards, as it can detect persistent scar tissue as well as right and left ventricular remodeling (44–46).

## SARS-CoV-2 vaccine-related myocarditis

### Prevalence and clinical presentation

There is significant epidemiological research and evidence on the reported adverse effects of myocarditis and pericarditis from these vaccines (47–53). The Center of Disease Control (CDC) in the US reported that cases of myocarditis were highest following the second dose of mRNA vaccination in young males (54). Patients usually present with chest pain, shortness of breath, fatigue, or palpitations, in order of prevalence (55, 56), with a temporal relationship to vaccine administration (52, 55, 57). The incidence remains low as seen in multiple studies and vaccine safety reports (28, 59), currently reported as between 0.5 and 2 per 100,000 people (54, 60).

Given the very high absolute number of vaccinations, however, this is and will remain a significant clinical problem.

### Proposed mechanisms

Among several proposed mechanisms for this injury are a dysregulated immune response (61), and activation of the complement system via immune complex formation involving anti-spike protein antibodies (62). Another potentially important mechanism proposed is a direct effect of vaccine nanoparticles on the myocardium, with a subsequent complement activation (63).

### Diagnostic utility of CMR

Most studies used the updated Lake Louise Criteria to diagnose myocarditis (3). CMR findings when summarized from multiple case series and original research articles report the severity of myocardial injury as mild (6, 52, 53, 57, 64–69). In several CMR studies on cardiac involvement in COVID-19, myocarditis was more prevalent than pericarditis. LGE was predominantly located in the inferior and inferolateral regions, subepicardial pattern and with co-located edema. Fronza et al. (70) and Groschel et al. (6), among others, described the regional distribution pattern of CMR findings, suggesting a basal/lateral predilection for irreversible injury. Fewer articles reported or mentioned pericarditis, which reported a lower prevalence of pericardial involvement with isolated pericarditis or co-located with myocardial LGE.

## Discussion

CMR as the non-invasive diagnostic standard in patients with myocardial involvement in systemic disease has revealed evidence for myocardial inflammation and inflammatory injury in patients with acute COVID-19 or thereafter. This is of clinical importance as such injury may occur in less severe cases and still cause persisting symptoms and impair prognosis. In fact, patients with acute, chronic, or post-vaccination disease all appear to present with similar symptoms, most often non-typical chest pain, shortness of breath, palpitations, and fatigue. Of note, most studies have not found a significant relationship between symptoms and CMR findings (6, 71, 72).

In many post-COVID patients, regional or global myocardial edema can be found (32, 73–75). The typical distribution is a non-ischemic pattern with subepicardial scarring inferior and inferolateral, mostly basal and mid-ventricular. A few studies reported intra-myocardial injury in vaccine-related myocarditis compared to the other 2 groups with a more subepicardial pattern (6). Pericardial effusion and pericarditis were less common. In our experience, pericardial effusion, however, is often localized (mostly lateral-basal) and thus may have been missed in previous studies.

Groschel et al. (6) recently compared CMR findings between the three syndromes and found a higher global T1 in the post-COVID group compared to controls, and a higher basal T1 in the post-COVID and COVID vaccination group. The group with myocardial involvement after vaccination also had a higher segmental involvement rate. No statistical difference was found in myocardial T2 and ECV between the groups, but global T2 values between post-COVID and controls were significant. The most common regions were basal and midventricular, lateral, inferior, or inferolateral, with a similar distribution frequency among the groups. The authors speculated that the difference in higher T1 and lower T2 times could be confounded by age, BMI, or weight (76, 77).

**Figure 1:**
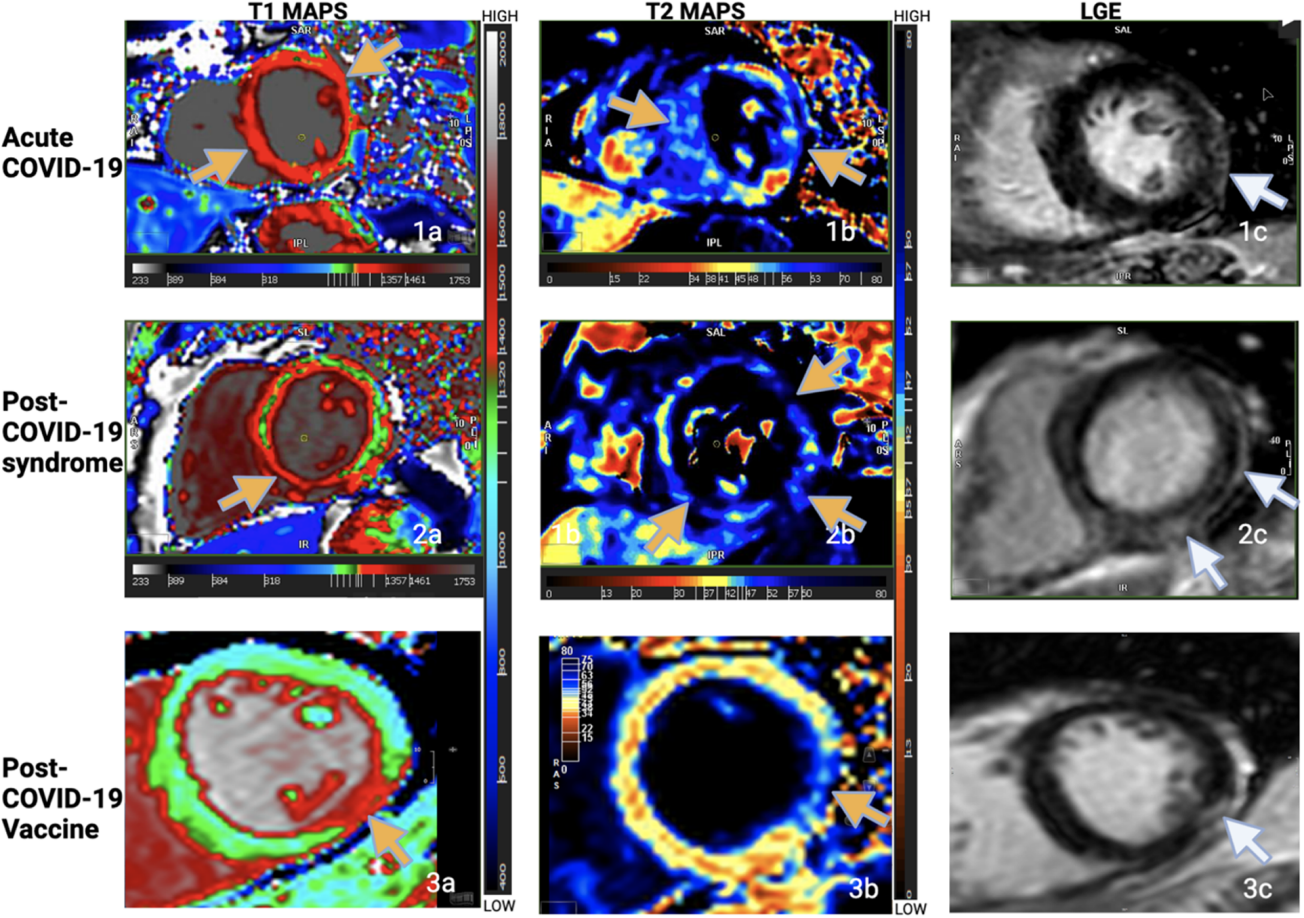
T1 maps, T2 maps, LGE imaging from one patient from each of the groups. Increased T1 and T2 times in the maps as shown by the yellow arrows. Subepicardial lateral or inferolateral scar in the basal or mid slices as shown by the white arrows. Note the pattern and location of myocardial injury in all three groups. (Figure adapted from the abstract poster by Garg et al. “Comparison of CMR Findings in Symptomatic Patients with Different COVID-Related Syndromes”, SCMR 25th annual scientific sessions, 2022. I am an author of this article; the conference grants authors the right to reuse their own figures without permission in future work.).

Summarizing the literature, there is conflicting data from studies about inflammation and results for myocardial T1 and T2, likely because the duration between illness and CMR was variable, representing different stages of the disease. Furthermore, the composition of the studied patient populations was variable, some with in-patients or ICU admissions, others with out-patient settings. Moreover, symptom burden and proximity of CMR to the presence of symptoms were also variable. Finally, patient demographics and co-morbidities varied widely. The similarity in the scar pattern amongst the three groups (6, 67) as also seen in this case series at our center (67), with co-located edema and LGE, however, suggests a common pathophysiology (Figure 1).

As explained in the subsections above, the underlying pathophysiology for cardiac injury in the three COVID-related conditions may reflect endotheliitis as direct injury and a systemic cytokine storm as indirect injury in the acute-COVID group, a compensatory anti-inflammatory response in post-COVID, and a dysregulated immune response in the COVID-vaccine group.

Endotheliitis, caused by either direct viral entry or activation of complement cascade may be a potential explanation. This endothelial injury may lead to microvascular dysfunction explaining the symptoms and CMR findings in these patients.

Several studies have studied microvascular dysfunction in acute COVID-19 (78, 79), post COVID (80–82), and post-COVID-vaccine (83) groups. This possibly common pathophysiology needs further exploration with methods such as stress CMR or novel non-invasive techniques like oxygenation-sensitive CMR (84).

The published evidence is still incomplete and subject to several limitations.

In most studies, only symptomatic patients were studied. Biopsy data are scarce, and while CMR is the de facto modality of choice and widely used for diagnosing myocarditis, endomyocardial biopsy (EMB) is still by many considered the gold standard. Most of the time, CMR findings are not corroborated with EMB because of the invasive nature of the latter and current guidelines that restrict it to specific, more severe cases. T1 and T2 values may vary between scanners and are therefore not generalizable between different centers or scanners. Furthermore, as mentioned above, reading CMR images requires expertise and are subject to inter-observer variability, likely explaining the varying prevalence of myocarditis between published cohorts. In athletes, regional fibrosis at the insertion points of the right ventricle, a non-specific finding, may be misinterpreted as inflammatory and lead to an overestimation of the incidence of myocardial involvement. Finally, abnormal LGE represents irreversible injury regardless of its stage. It could be acute, but also reflect such an insult years ago. Therefore, studies confined to LGE or T1 mapping lack information on acute or active inflammation (84).

In summary, CMR studies indicate a similarity of myocardial injury patterns between acute disease, post-COVID, or SARS-CoV-2 vaccination, suggesting a non-specific pathophysiology. It is therefore plausible that most instances of myocardial inflammation stem from generic inflammatory injury rather than direct viral injury. From a clinical standpoint, the exact underlying mechanism, however, is only partly significant. Barring rare cases of viral persistence (which is improbable in COVID), CMR-confirmed myocardial inflammatory injury (necrosis in a non-ischemic regional distribution) accompanied by edema as an active inflammation marker suffices for informing clinical decision-making.

Alas, therapeutic options for acute myocardial inflammation are limited. To combat COVID-related myocardial injury, we must develop better, personalized immune modulation strategies. The ball in the game against COVID-related myocardial injury is in the therapy court.
